# Super‐Resolution Spatial Proximity Detection with Proximity‐PAINT

**DOI:** 10.1002/anie.202009031

**Published:** 2020-11-09

**Authors:** Florian Schueder, Juanita Lara‐Gutiérrez, Daniel Haas, Kai Sandvold Beckwith, Peng Yin, Jan Ellenberg, Ralf Jungmann

**Affiliations:** ^1^ Faculty of Physics and Center for Nanoscience LMU Munich Geschwister-Scholl-Platz 1 80539 Munich Germany; ^2^ Max Planck Institute of Biochemistry Am Klopferspitz 18 82152 Martinsried Germany; ^3^ Department of Systems Biology and Wyss Institute for Biologically Inspired Engineering Harvard Medical School 3 Blackfan Circle Boston MA 02115 USA; ^4^ Cell Biology and Biophysics Unit European Molecular Biology Laboratory (EMBL) Meyerhofstraße 1 69117 Heidelberg Germany

**Keywords:** DNA-PAINT, Protein-Protein interactions, Proximity detection, Single Molecule Imaging, Super-resolution microscopy

## Abstract

Visualizing the functional interactions of biomolecules such as proteins and nucleic acids is key to understanding cellular life on the molecular scale. Spatial proximity is often used as a proxy for the direct interaction of biomolecules. However, current techniques to visualize spatial proximity are either limited by spatial resolution, dynamic range, or lack of single‐molecule sensitivity. Here, we introduce Proximity‐PAINT (pPAINT), a variation of the super‐resolution microscopy technique DNA‐PAINT. pPAINT uses a split‐docking‐site configuration to detect spatial proximity with high sensitivity, low false‐positive rates, and tunable detection distances. We benchmark and optimize pPAINT using designer DNA nanostructures and demonstrate its cellular applicability by visualizing the spatial proximity of alpha‐ and beta‐tubulin in microtubules using super‐resolution detection.

## Graphical abstract

A modified implementation of DNA‐PAINT microscopy is used to detect spatial proximity of biomolecules with super‐resolution capabilities. The new technique, called Proximity‐PAINT, features a precisely tunable detection range, high sensitivity and low false‐positive rates. The implementation can be applied to visualize cellular protein‐protein interactions and other biomolecules of interest, such as nucleic acids.

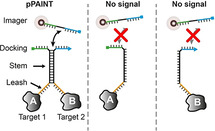

The coordination of the myriad of processes occurring within a cell relies on direct interactions among their molecular components, such as nucleic acids and proteins. In order to understand life on the molecular level, it is thus paramount to develop techniques that are able to visualize and quantify proximity of biomolecules. For example, mechanisms that regulate protein activity and their structural arrangement require components to be in close spatial proximity.^[1]^ Furthermore, knowledge about the precise location of these interactions within a cell could yield fundamental information about the underlying molecular mechanisms. Over the last decades, multiple techniques have been developed to interrogate the existence of protein‐protein interactions (PPI′s).^[2]^ However, most approaches fail to provide the spatial context of PPI′s and often depend on genetic and biochemical methods that rapidly increase complexity.

Imaging‐based methods, on the other hand, offer the advantage of spatially resolved characterization of PPIs in the native context of a cell. Chief among such techniques is Förster Resonance Energy Transfer (FRET), which allows sensitive distance measurements between two molecules of interest.^[3]^ However, the working range of FRET is traditionally limited to a few nanometers and quantitative distance readouts are challenging due to sensitivity to changes in the local dye environment (e.g. pH, ionic concentration, temperature).^[4]^ Recently, DNA‐based Proximity Ligation Assays (PLA) were developed, featuring rationally designed “logic AND gates” for the detection of proximity between two protein targets. In image‐based versions of PLA, a diffraction‐limited fluorescent signal is created via DNA amplification reactions, when two DNA strands (acting as proxies for protein targets) are ligated.^[5]^ However, the intrinsic amplification steps in classical PLA limits the possibility of detecting the precise sub‐diffraction localization of molecular proximity.

Here, we report the development of a versatile and programmable method for in situ proximity detection between two molecular targets with super‐resolution readout capability and call it Proximity‐PAINT (or pPAINT). The pPAINT approach is based on DNA‐PAINT^[6]^ super‐resolution microscopy. The transient binding of fluorescently labeled oligonucleotides (“imager” strands) in DNA‐PAINT produces the stochastic “blinking” of a subset of target molecules that can later be reconstructed to yield a super‐resolved image. To extend DNA‐PAINT for molecular proximity detection, we apply the same concept employed in split fluorescent proteins,^[7]^ where for example, GFP is split into two non‐fluorescent fragments, which can reform into a functional fluorescent protein when brought into close spatial proximity. This approach has been widely used to investigate PPI′s. Inspired by this, we split a classical DNA‐PAINT docking strand into two equal halves and used the fact that binding of a full‐length imager to either one of the halves would not be detectable due the highly reduced dwell times of this interaction. However, if the split DNA‐PAINT docking sites co‐localize, a binding signal would again be detectable, thus highlighting spatial proximity of two molecular targets. We rationally designed and quantitatively characterized pPAINT using designer DNA nanostructures,^[8]^ optimizing the system for highest detection efficiency while preventing false positive interactions. We furthermore implemented a concept to rationally tune the distance range of pPAINT from zero to tens of nanometers. Finally, we demonstrate pPAINT′s applicability to cellular protein proximity detection by visualizing the close association of alpha‐ and beta‐tubulin proteins in microtubules with high fidelity.

To faithfully detect the interaction between two targets of interest using pPAINT, each target is labeled with one of the two DNA strands that comprise the pPAINT system (Figure 1). If the target pair is in close proximity, the two split docking sites will spatially co‐localize, yielding a full, detectable DNA‐PAINT docking strand by the transient hybridization of a complementary stem (black sequence section in Figure 1 a). If one of the two targets is not within a desired spatial distance (tunable by a leash region, orange in Figure 1 a), or completely missing, no pPAINT signal should be detectable. Thus, pPAINT can effectively act as a “logic AND gate” that can be employed for in situ detection of proximity interactions at single‐molecule resolution. To rationally design and benchmark pPAINT′s performance and tunability, we employed designer DNA origami nanostructures,^[8]^ as precise nanobreadboards for arranging pPAINT strands at various spatial geometries and distances (Figure S1). In DNA origami, ≈200 short DNA oligomers (“staples”) are designed to be complementary to the sequence of a ≈7000 nucleotide long circular single‐stranded “scaffold”. Each staple has a unique sequence and specifically binds to parts of the scaffold, “folding” it into a pre‐designed shape.

**Figure 1:**
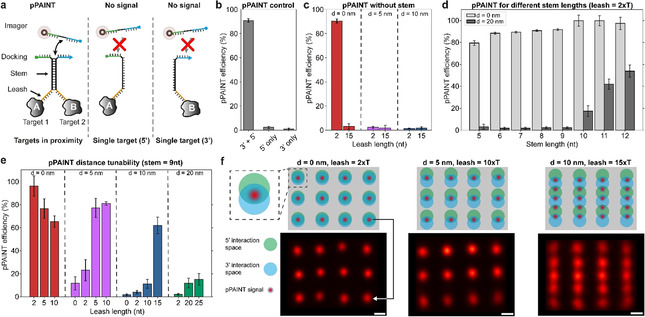
(a) pPAINT probes for spatial proximity detection of two targets using rationally designed DNA molecules. Each DNA strand features a leash (orange), stem (black) and half a DNA‐PAINT docking strand (green or blue). If two targets are in close proximity (tuneable by the length of the leash), a transient stem hybridizes, aligning both split strands to form a complete DNA‐PAINT docking site, yielding a positive pPAINT signal. If the targets are not within spatial proximity (or one is missing completely), binding times of the pPAINT imager to either split site are too short to be detected. (b) pPAINT proof‐of‐principle without leash or stem with both split sites directly adjacent (3′ and 5′), or one missing (5′ or 3′ only). (c) pPAINT distance dependency without stem for two leash lengths and three distances, indicating a stem necessity to achieve tuneable pPAINT distances. (d) Quantification of pPAINT false positive signals for different stem lengths. Combined leash and stem length should only allow pPAINT for *d*=0 nm distance, but not 20 nm, suggesting an ideal stem length between 9 and 10 nt under tested experimental conditions. (e) pPAINT detection distances can be tuned by modulating the leash length. (f) pPAINT super‐resolution proof‐of‐concept using designer 20‐nm‐grid DNA origami. Green and blue circles represent possible interaction radii of the 5′ and 3′ split docking strands. As expected, for 0‐nm, and 5‐nm spacing of pPAINT strands along the vertical axis, a 20‐nm‐grid pPAINT pattern can be observed (left and middle panel). For 10‐nm vertical spacing (right panel), more interaction partners become available, resulting in a 10‐nm‐spaced pattern along the vertical axis. A horizontal 20‐nm pattern is visible, again highlighting the distance control of our pPAINT implementation. Images represent summed localizations from ≈100 structures for each condition. Scale bars: 10 nm.

To quantify the detection sensitivity under different experimental conditions, we placed the pPAINT sensor (consisting of the two strands described above) at the center of the DNA origami (Figure S1). The edge of the origami was decorated with 52 DNA‐PAINT docking strands (orthogonal to the pPAINT site) resulting in a frame around the pPAINT site at the center. Using Exchange‐PAINT,^[6a]^ we first imaged the pPAINT sensor, followed by the frame. The signal of the frame was used to detect each origami, which was used as a reference region of interest for downstream pPAINT quantification (Figure S2, see Supporting Information for further details). We employed this workflow to characterize all pPAINT performance metrics such as false positives as well as optimal stem and leash lengths for distance tunability. DNA origami allowed us to gather quantitative results in a controlled manner, which would otherwise be hard to achieve.

In a first proof‐of‐principle experiment, we designed the pPAINT sensor without a stem or leash region in order to quantify pPAINT′s capability of detecting immediate spatial proximity with no spacing between the two split docking sites (Figure 1 b). To achieve this, we directly extended two adjacent staple strands in a DNA origami at the 3′‐ and 5′‐end by two T bases and the corresponding half of the split docking site. Using our benchmark assay, we detected a positive pPAINT signal in 91 % of all cases (we note that detection efficiencies are adjusted by respective incorporation efficiencies for staple strands in DNA origami,^[9]^ see Table S1 for further details). To ensure that this high detection efficiency is not an artifact of potential false positive signals, which might originate from solitary split docking strands, we performed experiments where only the 3′‐ or 5′‐extension was incorporated in our DNA origami platform, and detected negligible pPAINT signals in 3 % and 1 % of all cases for the 5′‐ and 3′‐extension, respectively (Figure 1 b and Table S2). Thus, the presence of only one of the half‐docking sites alone cannot produce a detectable signal, making the split docking site a robust system for proximity detection. Next, we explored the possibility of pPAINT with increased leash length from two to 15 nt (but still without the inclusion of a stem) to detect larger molecular distances of up to 10 nm (Figure 1 c and Table S3). Neither two nor 15 nt poly‐T leashes yielded any detectable pPAINT signal for split strands spaced 5 and 10 nm apart on the DNA origami platform. Interestingly, the 15 nt poly‐T leash did not show a positive pPAINT signal even for the 0‐nm distance, most likely due to the increased flexibility of the strands.

In order to probe larger molecular distances, a semi‐stable stem region was thus a necessity for pPAINT. However, the inclusion of a complementary stem region between the two halves of the pPAINT system could lead to false positives, if the stability of the stem would be increased to a point where it could force the two entities of the pPAINT sensor together, even in absence of spatial proximity of an underlying biomolecular system under investigation. In order to assay the stem stability with regards to possible false positives, we designed one DNA origami structure in which the two pPAINT entities were spaced 0 and 20 nm apart (Figure 1 d and Table S4). As the system is designed without a leash, any positive pPAINT signal originating from the 20‐nm distances would be false positives, mediated by an undesired stable stem interaction. While the true positive pPAINT signal at 0‐nm distance was similar for a stem length from 5 nt to 12 nt (Figure 1 d, light gray bars), false positives became apparent at a stem length of longer than 10 nt (Figure 1 d, dark gray bars).

Based on these results, we next probed pPAINT′s ability to detect proximity at different distances using a stem length of 9 nt (Figure 1 e and Table S5) and 10 nt (Figure S3 and Table S6). We evaluated four distances (0 nm, 5 nm, 10 nm and 20 nm) using three different leash lengths. pPAINT detection efficiency for 0‐nm distances decreased when the leash length was increased, again most likely due to increased flexibility. For finite spacings (5 nm, 10 nm, and 20 nm), the detection efficiency increased with leash length, highlighting the possibility of pPAINT to detect molecular proximity at different distances, tunable by a rationally designed leash. We note that for cases, where the distance to be measured is equal to the designed length of the leash, an additional base of the stem (e.g. 10 nt vs. 9 nt) will lead to a detectable difference in pPAINT efficiency, as the added base can effectively act as a leash extension. However, due to the potential of creating false positives, we suggest making use of only the leash length to tune the detection distance. Additionally, we note that detection efficiencies for a 10 nt stem increased in general due to the increased probability of co‐localizing the split docking sites (for all efficiency values see Supplementary Tables 2–6). As a final in vitro benchmark, we sought to demonstrate pPAINT′s capability to visualize molecular proximity at super‐resolution. To this end, we designed pPAINT sensors arranged in a 3×4 grid with a spacing of 20 nm on a DNA origami. The top of Figure 1 f schematically shows the DNA origami, where the green and blue circles represent the interaction radii of the 5′‐ and 3′‐half‐docking sites. When both circles overlap, the calculated leash length allows the formation of the pPAINT docking site (represented by a red point). The bottom of Figure 1 f shows the experimental results, represented by sum images of single DNA origami structures (≈100 structures for each condition, see Figures S4–6 for single structures). We designed three pPAINT patterns, where a total of 12 pPAINT pairs were spaced ≈20 nm horizontally and 0, 5, and 10 nm vertically (Figure 1 f left, middle, and right). The corresponding leash length was 2, 10, and 15 nt for the 0, 5, and 10 nm distance. In the case of 0 and 5 nm spaced interaction sites, a clear 20‐nm‐grid pattern was resolved as designed. The leash length of 15 nt used in the origami with 10 nm spacing allowed each probe strand to interact with two of its neighbors along the vertical axis. As a result of the transient interaction of the stem, the dual interaction possibility effectively turned this design into a 5×4 grid, with 10‐nm‐spaced pPAINT signals vertically. Importantly, the spacing between neighbors along the horizontal axis is ≈22 nm, thus precluding the possibility of interaction between neighboring probes in the horizontal axis, as seen in the resulting sum image in Figure 1 f, again highlighting the tunable interaction distance and low false positives of pPAINT.

Finally, we assessed pPAINT′s cellular applicability by targeting alpha‐ and beta‐tubulin in microtubules of fixed U2OS cells. We chose microtubules as cellular proof‐of‐concept system, as they are composed of alpha‐ and beta‐tubulin heterodimers with monomers spaced ≈4 nm apart in a well‐defined geometry. Furthermore, microtubules are one of the de‐facto standards in super‐resolution microscopy in terms of imaging performance comparison. In order to visualize each individual pPAINT strand separately, we furthermore incorporated DNA‐PAINT docking sites in the leash (Figure 2 a,f,k). To target alpha‐ and beta‐tubulin proteins, we chose primary and secondary antibodies, with the pPAINT strands conjugated to secondary antibodies. As these were not site‐specifically conjugated with DNA strands, we did not have complete control over the number of DNA strands per secondary antibody. To avoid unwanted multivalent interactions of stems, we modified our pPAINT sensor system featuring stem sequences, which allowed for transient intramolecular hairpin formation,^[10]^ alleviating potential stem‐induced co‐localization of pPAINT strands. As a negative control, we first incubated the sample without the primary antibody against alpha‐tubulin, but with both secondary antibodies present (Figure 2 a). This experimental design reliably exemplifies the conditions when two target proteins do not exhibit spatial proximity. As expected, the control DNA‐PAINT image targeting the P39 sequence leash yielded a super‐resolution signal of microtubules (Figure 2 b). The DNA‐PAINT image with imagers targeting the P5 sequence leash, on the orthogonal secondary antibody yielded no signal (as designed), highlighting the absence of the second pPAINT strand. The corresponding pPAINT round (Figure 2 d) showed no detectable signal, proving no false positives under these conditions. A final repeat of the P39 imaging round revealed that antibodies were still in place during all Exchange‐PAINT^[6a]^ rounds (Figure 2 e). The corresponding negative control experiments with missing primary antibodies against beta‐tubulin showed comparable results (Figures 2 f–j). Finally, when incubating both primary and secondary antibodies (Figure 2 k) against alpha‐ and beta‐tubulin (see Figure 2 l,m for leash controls), a positive pPAINT signal was detected (Figure 2 n), and the corresponding zoom‐in revealed high‐quality super‐resolution of microtubules (Figure 2 o).

**Figure 2:**
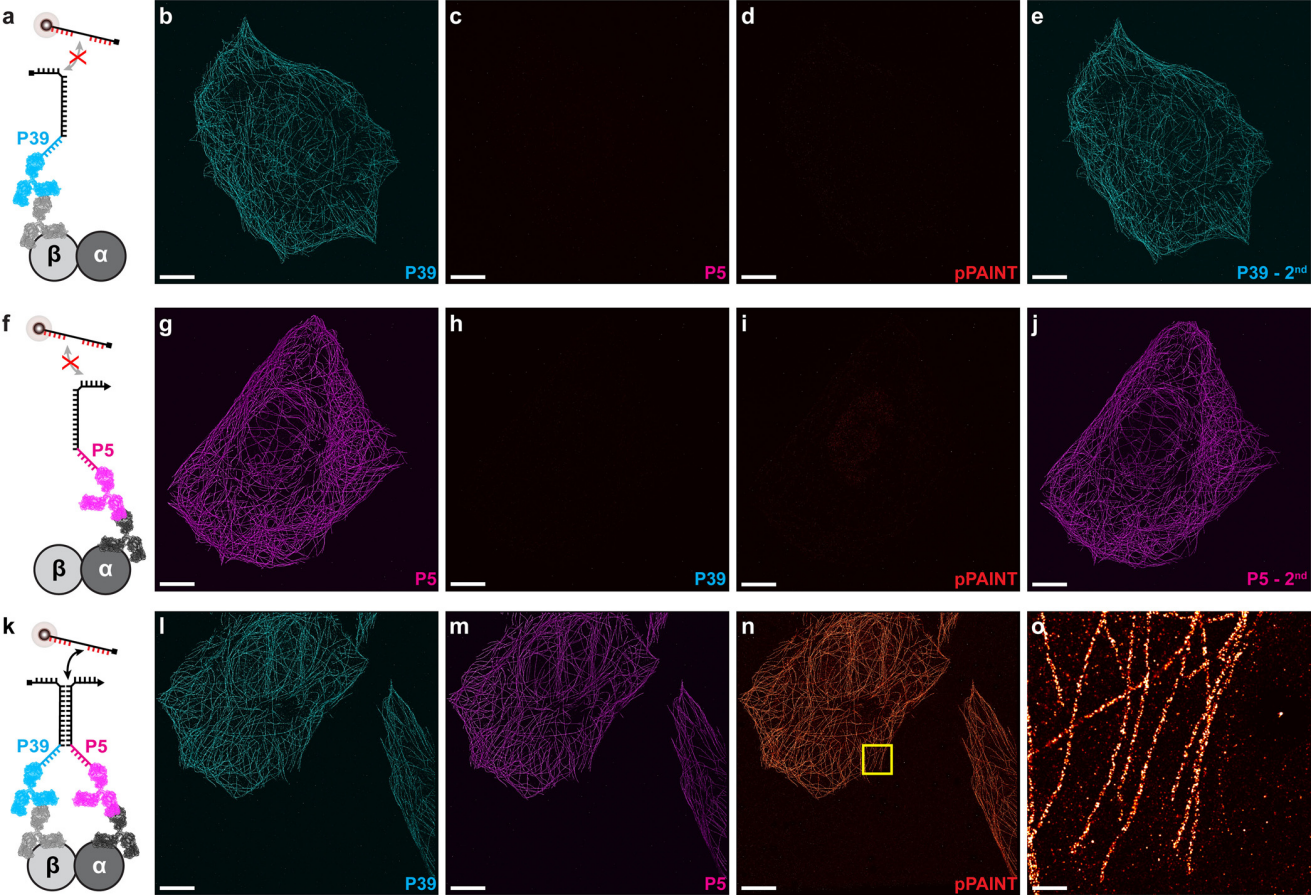
Alpha‐ and beta‐tubulin is targeted using primary and DNA‐conjugated secondary antibodies. pPAINT strands consist of P39 and P5 classical DNA‐PAINT docking strands as leashes to visualize correct protein targeting. (a) Negative control, where the sample is incubated with both secondary antibodies, however the primary antibody against alpha‐tubulin is missing. (b) DNA‐PAINT control using P39* imager yields a super‐resolved microtubule network. (c) Corresponding DNA‐PAINT image using P5* imager shows no signal. (d) pPAINT imaging shows no detectable signal. (e) Repeated P39* imaging shows similar results as in b, showing that antibodies have not dissociated. (f) Corresponding negative control where the primary antibody against beta‐tubulin is missing. (g–j) Corresponding experiments to c‐e show similar results. (k) Positive pPAINT experiment where all primary and secondary antibodies are incubated. (l,m) DNA‐PAINT control using P39* and P5*imager shows both secondary antibody signals are present. (n) Positive pPAINT supports its applicability in a cellular setting. (o) Zoom‐in of highlighted area in n shows high‐quality super‐resolution imaging of microtubules using pPAINT. Scale bars: 5 μm (b–e, g–j, l–n), 500 nm (o).

In conclusion, we have introduced pPAINT, a modular and programmable proximity detection assay based on split docking sites for DNA‐PAINT featuring tunable distance detection ranges and good detection efficiency with negligible false positives. We quantitatively assayed pPAINT′s performance using designer DNA origami structures and demonstrated its applicability in a cellular proof‐of‐concept. Our system underwent a careful characterization pipeline with the goal of engineering an assay that detects interacting protein pairs with both high sensitivity and accuracy. Using DNA origami as a precise breadboard, we proved that the tunable leash length sets an upper limit on the detection radius with nanometer precision. The stem was rationally designed to transiently bring together the strands and assemble a two‐component docking site for an imager that was specifically adapted for this application. The transient assembly of the pPAINT system is a crucial feature, as it precludes the binding of pPAINT probe pairs in solution, thus reducing the false positive rate. Furthermore, the transient nature of the stem binding allows the interaction of a pPAINT probe with neighboring pPAINT sites, as long as their interaction radii intersect. This feature can be exploited to identify distinct interacting protein pairs within a multimeric group of proteins: each half of the pPAINT imager can encode the identity of a protein species and the complete imager thus encodes the identity of a protein pair. Furthermore, qPAINT^[11]^ could be used to perform stoichiometric interaction analyses, quantifying interaction frequencies of different pPAINT pairs. Novel affinity binders such as nanobodies,^[12]^ affimers,^[13]^ and aptamers^[14]^ could be employed to efficiently label proteins with 1:1 stoichiometry, improving pPAINT′s cellular performance. Interestingly, nanobodies that target different epitopes within the same protein could be used to trace the conformational changes of a protein and its association with other proteins of the same type (e.g. homodimers). Finally, pPAINT could be extended in a straightforward manner to detect spatial proximity of proteins and nucleic acids for example, in a cell's nucleus.

## Conflict of interest

A patent application has been filed. P.Y. and R.J. are cofounders of Ultivue, Inc. R.J. is cofounder of Massive Photonics GmbH.

## Supporting information

As a service to our authors and readers, this journal provides supporting information supplied by the authors. Such materials are peer reviewed and may be re‐organized for online delivery, but are not copy‐edited or typeset. Technical support issues arising from supporting information (other than missing files) should be addressed to the authors.

Supplementary

Supplementary
