# Supplementary material for: Super‐Resolution Spatial Proximity Detection with Proximity‐PAINT
Source: Angew Chem Int Ed Engl. 2020 Nov 9;60(2):716–20. doi: 10.1002/anie.202009031 (PMC7839522; doi:10.1002/anie.202009031)
Supplement: Supplementary file 1 — Supplementary [file ANIE-60-716-s001.pdf]

Angewandte Chemie
Eine Zeitschrift der Gesellschaft Deutscher Chemiker

Supporting Information

Super-Resolution Spatial Proximity Detection with Proximity-PAINT

*Florian Schueder[+], Juanita Lara-Gutiérrez[+], Daniel Haas, Kai Sandvold Beckwith, Peng Yin, Jan Ellenberg, and Ralf Jungmann**

anie_202009031_sm_miscellaneous_information.pdf
anie_202009031_sm_xlsx.zip

Table of Contents

Supplementary Figure 1	DNA origami designs
Supplementary Figure 2	Quantitative workflow
Supplementary Figure 3	Quantitative distance measurements
Supplementary Figure 4	20 nm grids for pPAINT d=0nm
Supplementary Figure 5	20 nm grids for pPAINT d=5nm
Supplementary Figure 6	20 nm grids for pPAINT d=10nm
Supplementary Table 1	DNA origami staples extended for quantitative experiments
Supplementary Table 2	Quantitative pPAINT measurements (corresponding to Fig. 1b)
Supplementary Table 3	Quantitative pPAINT measurements Stem= 0 nt (corresponding to Fig. 1c)
Supplementary Table 4	Quantitative pPAINT measurements leash length = 2xT (corresponding to Fig. 1d)
Supplementary Table 5	Quantitative pPAINT measurements Stem = 9nt (corresponding to Fig. 1e and Figure S6)
Supplementary Table 6	Quantitative pPAINT measurements Stem = 10nt (corresponding to Figure S6)
Supplementary Table 7	pPAINT 3’ part
Supplementary Table 8	pPAINT 5’ part
Supplementary Table 9	Imager sequences
Supplementary Table 10	List of biotinylated DNA staple strands
Supplementary Table 11	Handle sequences
Supplementary Table 12	Imaging parameters
Supplementary References	

Experimental Procedures

Materials. Unmodified DNA oligonucleotides and biotinylated DNA oligonucleotides were purchased from IDT. Cy3B-modified DNA oligonucleotides were custom-ordered from Metabion. M13mp18 scaffold was obtained from Tilibit. Tris 1 M pH 8.0 (cat: AM9856), EDTA 0.5 M pH 8.0 (cat: AM9261), Magnesium 1 M (cat: AM9530G) and Sodium chloride 5 M (cat: AM9759) were obtained from Ambion. Ultrapure water (cat: 10977-035) was purchased from Gibco. Streptavidin (cat: S-888) was purchased from Thermo Fisher. BSA-Biotin (cat: A8549) was obtained from Sigma-Aldrich. Coverslips (cat: 0107032) and glass slides (cat: 10756991) were purchased from Marienfeld and Thermo Fisher. Double-sided tape (cat: 665D) was ordered from Scotch. Two component silica twinsil speed 22 (cat. 1300 1002) was ordered from picodent. 8-well flow chambers µ-Slide VI$^{0.5}$ were purchased from ibidi. Tween 20 (cat: P9416-50ML), glycerol (cat: 65516-500ml), methanol (cat: 32213-2.5L), protocatechuate 3,4-dioxygenase pseudomonas (PCD) (cat: P8279), 3,4-dihydroxybenzoic acid (PCA) (cat: 37580-25G-F) and (+−)-6-hydroxy-2,5,7,8- tetra-methylchromane-2-carboxylic acid (Trolox) (cat: 238813-5 G) were ordered from Sigma. PO (cat: P4234-250UN), C (cat: C40-100MG) and Glucose (G5767-25G) were ordered from Sigma-Aldrich. Potassium chloride (cat: 6781.1) was ordered from Carl Roth. Sodium hydroxide (cat: 31627.290) was purchased from VWR. McCoy's 5A medium (cat: 16600082) was ordered from Thermo Fisher Scientific. Fetal Bovine Serum (FBS) (cat: 10500-064), 1× Phosphate Buffered Saline (PBS) pH 7.2 (cat: 20012-019), 0.05 % Trypsin–EDTA (cat: 25300-054) and were purchased from Thermo Fisher Scientific. Glass-bottomed 8-well slides (cat: 0030742036) were ordered from Eppendorf. Falcon tissue culture flasks (cat: 734-0965) were ordered from VWR. Paraformaldehyde (cat: 15710) and glutaraldehyde (cat: 16220) were obtained from Electron Microscopy Sciences. Bovine serum albumin (cat: A4503-10G) was ordered from Sigma-Aldrich. Triton X-100 (cat: 6683.1), Sodium borohydride > 97 % (cat: 4051.1) was purchased from Roth. Monoclonal antibodies against alpha-tubulin (used: 1:200) (cat: MA1-80017) were purchased from Thermo Scientific. Monoclonal antibodies against beta-tubulin (cat: #2128) were obtained from Cell Signaling Technology (dilution 1:200). Secondary antibodies anti-rat (cat: 712-005-150) (used in a 1:100 dilution) and anti-rabbit (cat: 711-005-152) (used in a 1:100 dilution), were purchased from Jackson ImmunoResearch. 90 nm diameter Gold Nanoparticles (cat: G-90-100) were ordered from cytodiagnostics.

Buffers. Five buffers were used for sample preparation and imaging: Buffer A (10 mM Tris-HCl pH 7.5, 100 mM NaCl, 0.05 % Tween 20, pH 7.5); buffer B (10 mM $MgCl_2$, 5 mM Tris-HCl pH 8, 1 mM EDTA, 0.05 % Tween 20, pH). For the experiments in **Figure 1b-e** and **Figure S3** the corresponding imaging buffer was supplemented with: 1× Trolox, 1× PO, 1× C and 0.8 % G. For the experiments in **Figure 1f, Figures 2** and **Figures S4-S6** the corresponding imaging buffer was supplemented with: 1× Trolox, 1× PCA and 1× PCD (see paragraph below for details). Both photostabilization systems allowed us to maximize the number of photons per event and thus achieve optimal spatial resolution.

Trolox, PCA and PCD. 100× Trolox: 100 mg Trolox, 430 µl 100 % Methanol, 345 µl 1M NaOH in 3.2 ml H_2O. 40× PCA: 154 mg PCA, 10 ml water and NaOH were mixed and pH was adjusted 9.0. 100× PCD: 9.3 mg PCD, 13.3 ml of buffer (100 mM Tris-HCl pH 8, 50 mM KCl, 1 mM EDTA, 50 % Glycerol). All three were frozen and stored at -20 °C.

PO, C and G. 100× PO solution consists of 26 mg of PO in 684 µL of enzyme buffer (10mM Tris pH7.5, 50mM KCl, 20% Glycerol); 100× C solution consists of 2 mg Catalase in 1 ml enzyme buffer (10mM Tris pH7.5, 50mM KCl, 20% Glycerol). 50× G solution consists of 800 mg Glucose (G) in 2 ml water. All three were flash frozen in liquid nitrogen and stored at −80 °C.

DNA origami self-assembly. All DNA origami structures were designed with the Picasso[1] design tool (see **Figure S1**). Self-assembly of DNA origami was accomplished in a one-pot reaction mix with 50 µl total volume, consisting of 10 nM scaffold strand (sequence see **Data S1**), 100 nM folding staples (**Data S2-S4**), 10 nM biotinylated staples (**Table S10**), and 1 µM of docking site strands (List of DNA-PAINT handles see **Table S7 & S8**) in folding buffer (1× TE buffer with 12.5 mM $MgCl_2$). The reaction mix was then subjected to a thermal annealing ramp using a thermocycler. The reaction mix was first incubated at 80 °C for 5min and then immediately cooled down to 60 ºC. Subsequently, the sample was cooled from 60 to 4 °C in steps of 1 °C per 3.21 min and then held at 4 °C.

DNA origami sample preparation. For sample preparation of **Figure 1b-e** and **Figure S3**, a µ-Slide VI$^{0.5}$ from ibidi was used as sample chamber. First, 100 µl of biotin labeled bovine albumin (1 mg/ml, dissolved in buffer A) was flushed into the chamber and incubated for 5 min. The chamber was then washed with 500 µl of buffer A. A volume of 100 µl of streptavidin (0.5 mg/ml, dissolved in buffer A) was then flushed through the chamber and allowed to bind for 5 min. After washing with 500 µl of buffer A and subsequently with 500 µl of buffer B, 100 µl of biotin labeled DNA structures (~200 pM) in buffer B were flushed into the chamber and incubated for 8 min. The chamber was washed with 500 µl of buffer B. Finally, 100 µl of the imager solution in the corresponding imaging buffer (see **Table S12**) was flushed into the chamber.
For sample preparation of **Figure 1f** and **Figure S4-S6**, a piece of coverslip and a glass slide were sandwiched together by two strips of double-sided tape to form a flow chamber with inner volume of ~20 µl. First, 20 µl of biotin labeled bovine albumin (1 mg/ml, dissolved in buffer A) was flushed into the chamber and incubated for 2 min. The chamber was then washed with 40 µl of buffer A. A volume of 20 µl of streptavidin (0.5 mg/ml, dissolved in buffer A) was then flushed through the chamber and allowed to bind for 2 min. After washing with 20 µl of buffer A and subsequently with 20 µl of buffer B, 20 µl of biotin labeled DNA structures (~200 pM) in buffer B were flushed into the chamber and incubated for 2 min. The chamber was washed with 40 µl of buffer B. Finally, 20 µl of the imager solution in the corresponding imaging buffer (see **Table S12**) was flushed into the chamber, which was subsequently sealed with two component silica before imaging.

Antibody conjugation. Antibodies were conjugated to DNA-PAINT docking sites via maleimide-PEG2-succinimidyl ester chemistry as previously reported[1].

Cell culture. U-2 OS-CRISPR-Nup96-mEGFP cells were passaged every other day and used between passage number 5 and 20. The cells were maintained in McCoy's 5A medium supplemented with 10 % Fetal Bovine Serum. Passaging was performed using 1× PBS and Trypsin-EDTA 0.05 %. 24 h before immunostaining, cells were seeded on Eppendorf 8-well glass coverslips at 30,000 cells/well.

Cell fixation. For fixation, the samples were fixed and permeabilized with 3 % formaldehyde, 0.1 % glutaraldehyde and 0.25 % Triton X-100 for 12 min. Next, samples were rinsed twice (5 min) with 1× PBS and then quenched with 0.1 % NaBH4 for 7 min. After rinsing four times with 1× PBS for 30 s, 60 s, and twice for 5 min, samples were blocked and permeabilized with 3 % BSA and 0.25 % Triton X-100 for 2 h. Then, samples were incubated with 10 µg/ml of primary antibodies (1:200 dilution) in a solution with 3 % BSA and 0.1 % Triton X-100 at 4 °C overnight. Cells were washed three times (5 min each) with 1× PBS. Next, they were incubated with 10 µg/ml of labeled secondary antibodies (1:100 dilution) in a solution with 3 % BSA and 0.1 % Triton X-100 at room temperature for 2 hours. For fiducial based drift correction, the samples were incubated with gold nanoparticles with a 1:1 dilution in 1× PBS for 5 min. Finally, samples were rinsed three times with 1× PBS before adding imager solution.

Super-resolution microscope. Fluorescence imaging was carried out on an inverted microscope (Nikon Instruments, Eclipse Ti2) with the Perfect Focus System, applying an objective-type TIRF configuration with an oil-immersion objective (Nikon Instruments, Apo SR TIRF 100×, NA 1.49, Oil). A 561 nm (MPB Communications Inc., 2 W, DPSS-system) laser was used for excitation. The laser beam was passed through cleanup filters (Chroma Technology, ZET561/10) and coupled into the microscope objective using a beam splitter (Chroma Technology, ZT561rdc). Fluorescence light was spectrally filtered with an emission filter (Chroma Technology, ET600/50m and ET575lp) and imaged on a sCMOS camera (Andor, Zyla 4.2 Plus) without further magnification, resulting in an effective pixel size of 130 nm (after 2×2 binning).

Imaging conditions

Figure 1b-e. First round of imaging was carried out using an imager strand concentration of 7.5 nM (pPS) and 2.5 nM (P3) in imaging buffer (see **Table S12**). 20,000 frames were acquired at 100 ms exposure time. The readout bandwidth was set to 200 MHz. Laser power (@561 nm) was set to 20 mW (measured before the back focal plane (BFP) of the objective), corresponding to 113 W/cm^2 at the sample plane. After imaging the sample was subsequently washed five times with 100 µl each with 1× PBS (on the microscope). Second round of imaging was carried out using an imager strand concentration of 2.5 nM (P3) and 2.5 nM (P6) in imaging buffer (see **Table S12**). 5,000 frames were acquired at 100 ms exposure time. The readout bandwidth was set to 200 MHz. Laser power (@561 nm) was set to 100 mW (measured before the back focal plane (BFP) of the objective), corresponding to 564 W/cm^2 at the sample plane.

Figure 1f. Images were acquired with an imager strand concentration of 5 nM (pPS) in imaging buffer (see **Table S12**). 20,000 frames were acquired at 300 ms exposure time. The readout bandwidth was set to 200 MHz. Laser power (@561 nm) was set to 100 mW (measured at the back focal plane (BFP) of the objective). corresponding to 564 W/cm^2 at the sample plane.

Figure 2. Images were acquired with an imager strand concentration of 2 nM (pPJL), 0.3 nM (P5) and 0.3 nM (P39) in imaging buffer (see **Table S12**). 20,000 frames were acquired at 50 ms exposure time and a readout bandwidth of 200 MHz. Laser power (@560 nm) was set to 90 mW (measured before the back focal plane (BFP) of the objective), corresponding to 508 W/cm^2 at the sample plane.

Image analysis. Raw fluorescence data was subjected to spot-finding and subsequent super-resolution reconstruction using the 'Picasso' software package[1]. x, y and z drift correction was performed with a redundant cross-correlation and DNA origami or gold particles as fiducials.

Quantitative analysis. pPAINT data (**Figure 1b-e, Figure S3**) was selected based on the method described in **Figure S2**. The data was linked (gap size = 4 frames), and afterwards filtered. As filter criteria the 'mean frame' with 10% around the maximum, the 'std frame' >10% and the number of localizations with 15 < 'n events' < 75 were applied.

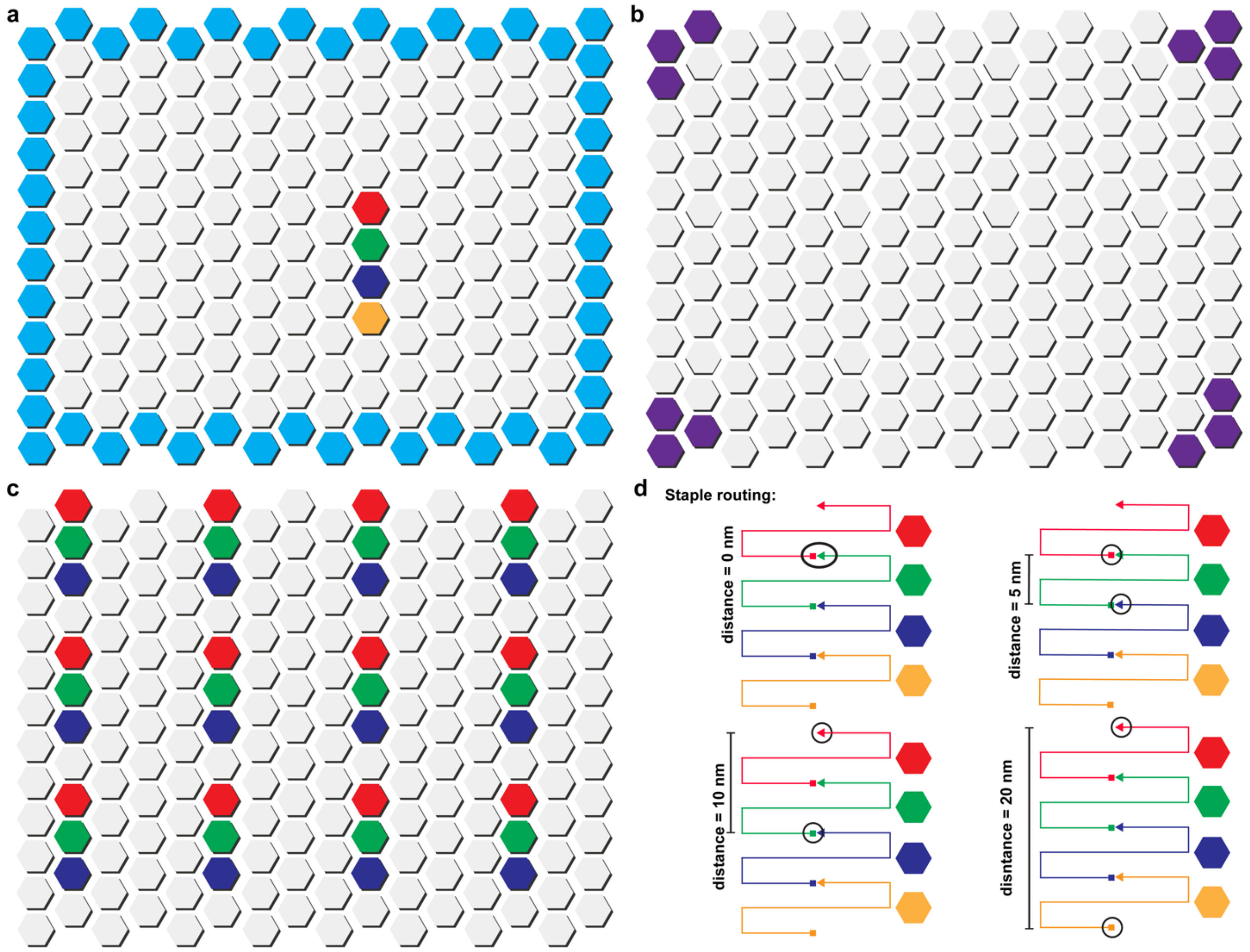

Figure S1. DNA origami designs. (a) DNA origami structure for quantitative pPAINT analysis (**Figure 1b-e, Figure S3**). The blue frame indicates 3'-extended staples with the P6 DNA-PAINT sequence. Red, green, dark-blue and yellow positions indicate pPAINT extensions for different distances (see **Panel d** and **Data S2** for staple sequences). **(b)** Four-corner DNA origami design. The purple positions indicate P3 DNA-PAINT sequence extensions (see **Data S3** for staple sequences). **(c)** 20-nm-grid design (**Figure 1f, Figure S4-S6**). Red, green and dark-blue position indicate pPAINT extensions for different distances (see **Panel d** and **Data S4** for staple sequences). **(d)** Staple routing for DNA origami in **Panel (a)** and partly in **Panel (c)**. Black circles indicate the staple and the site of extension (3' or 5') for a certain distance.

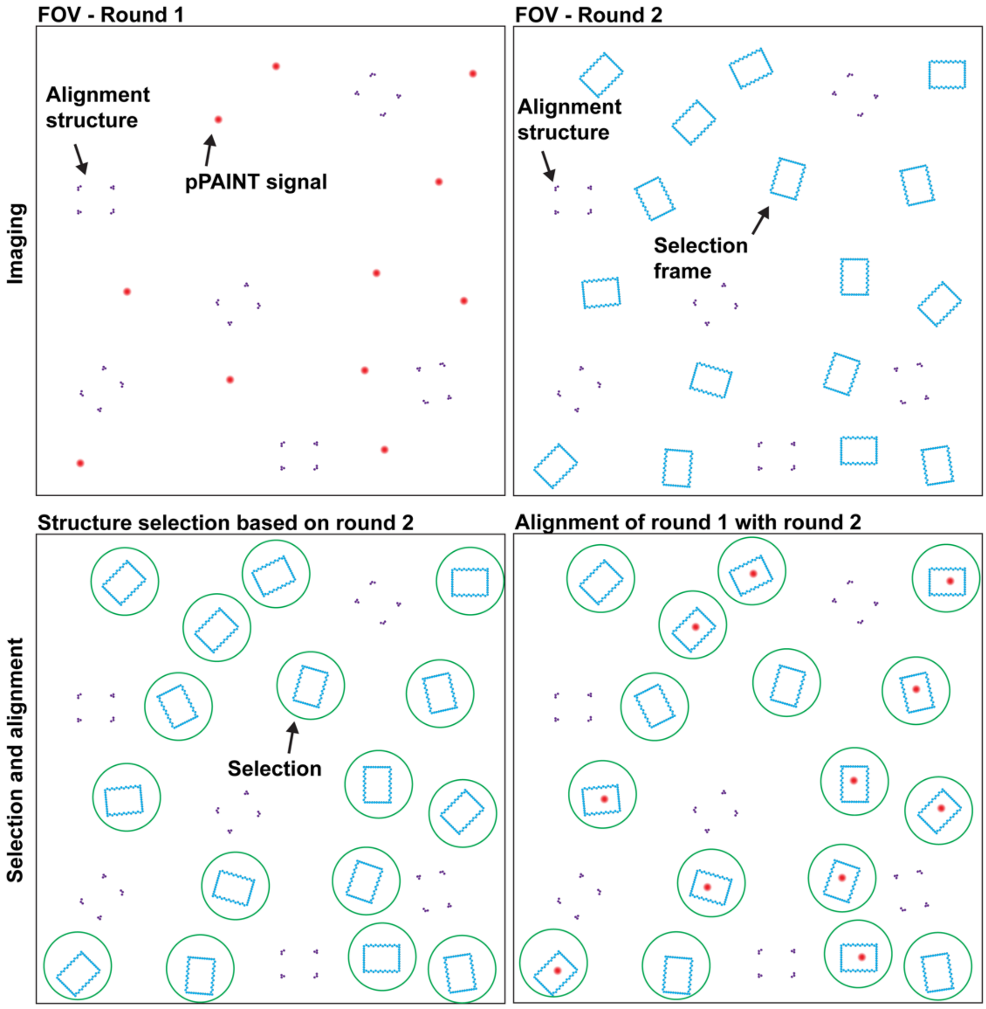

Figure S2. Quantitative workflow. In the first round, pPAINT (pPS) and four-corner structures (P3) (for later overlay and alignment) are imaged. In the second round the frame (P6) of the pPAINT origami and again the four corners (P3) are imaged. After super-resolution processing (localizing and drift correction) all pPAINT structures are selected ('picked') via the frame (round 2). Next, round 1 and round 2 are overplayed and aligned (via four-corner structures) with each other. Finally, all selected structures (based on the frame selection from round 2) are analyzed for pPAINT signal. Based on this analysis, a quantitative detection yield for pPAINT can be calculated.

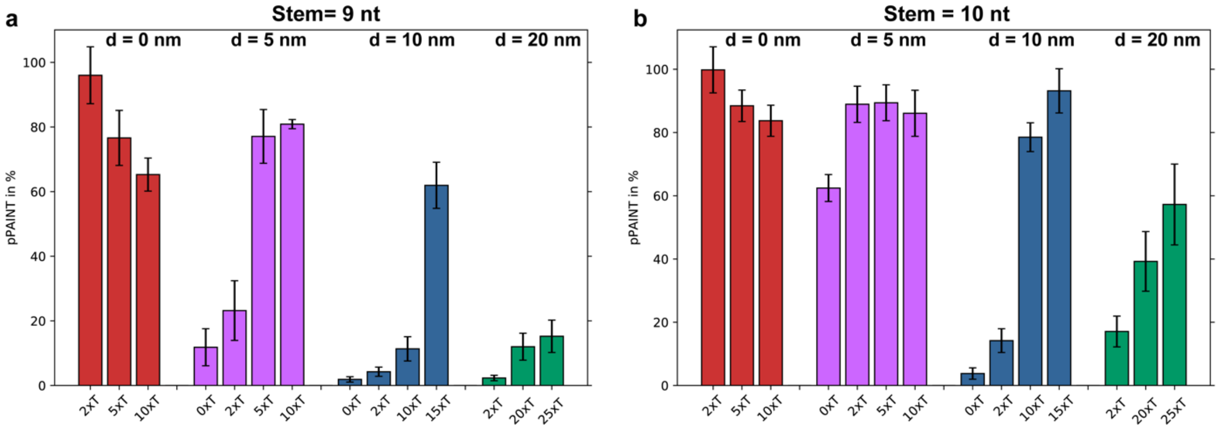

Figure S3. Quantitative distance measurements. (a) Distance measurements for d = 0 nm (red), d = 5 nm (magenta), d = 10 nm (blue) and d = 20 nm (green) for different leash lengths (poly-T, x-axis) and a stem of 9 nt (see **Table S5**). **(b)** Distance measurements for d = 0 nm (red), d = 5 nm (magenta), d = 10 nm (blue) and d = 20 nm (green) for different leash lengths (poly-T, x-axis) and a stem of 10 nt (see **Table S6**).

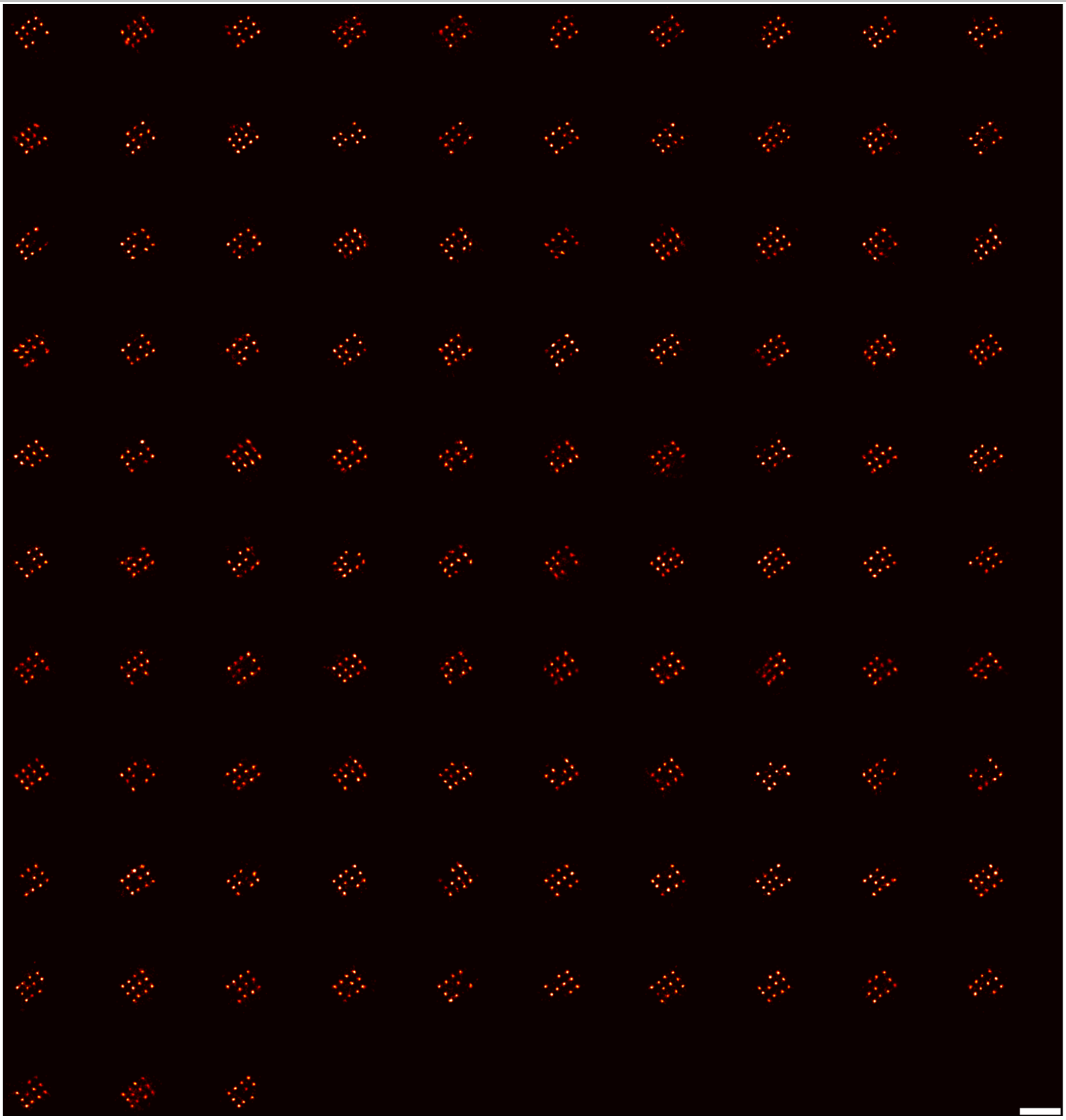

Figure S4. 20-nm-grids for pPAINT d=0nm (corresponding to **Figure 1e** (left)). Scale bar 100 nm.

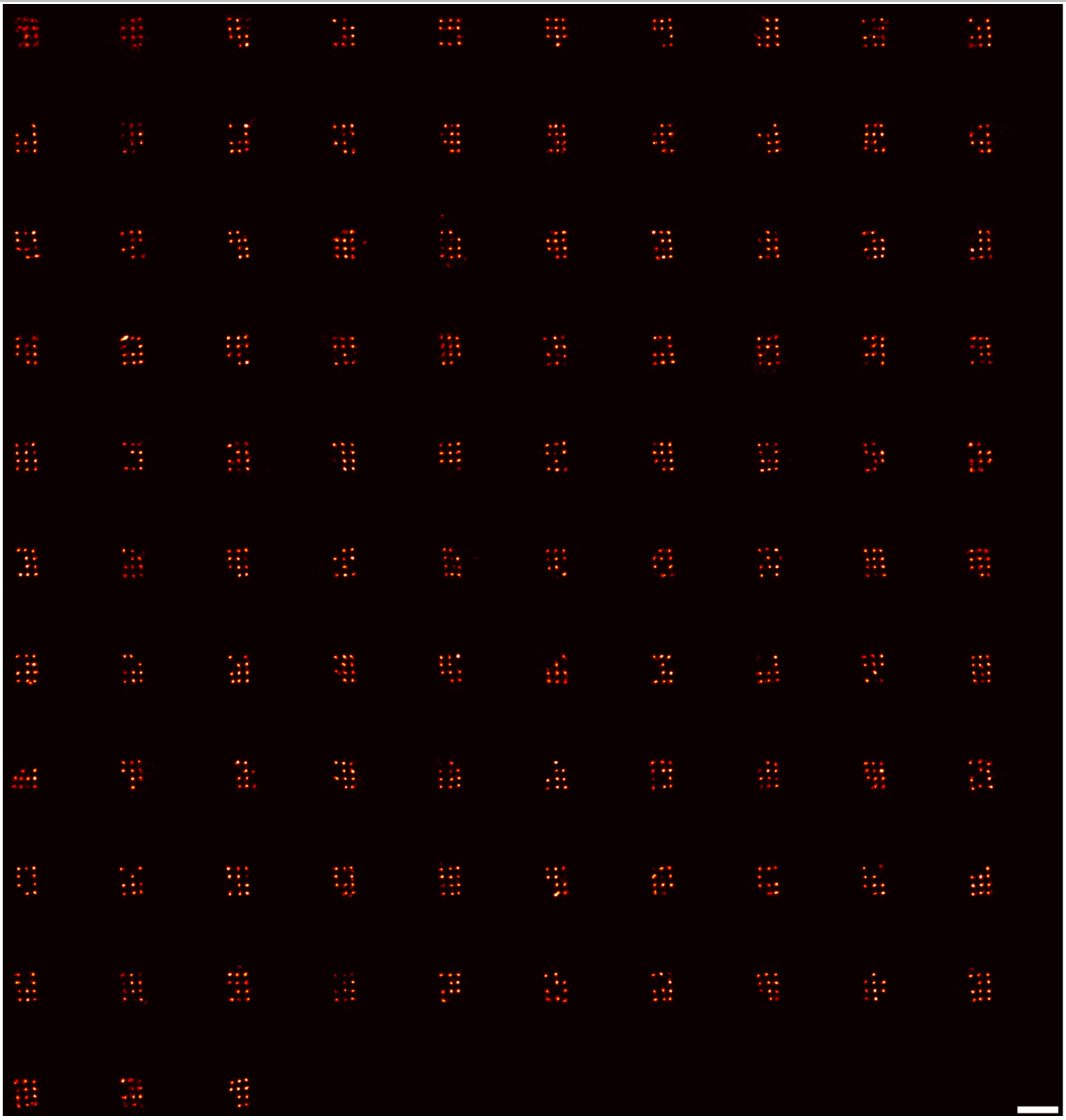

Figure S5. 20-nm-grids for pPAINT d=5nm (corresponding to **Figure 1e** (middle)). Scale bar 100 nm.

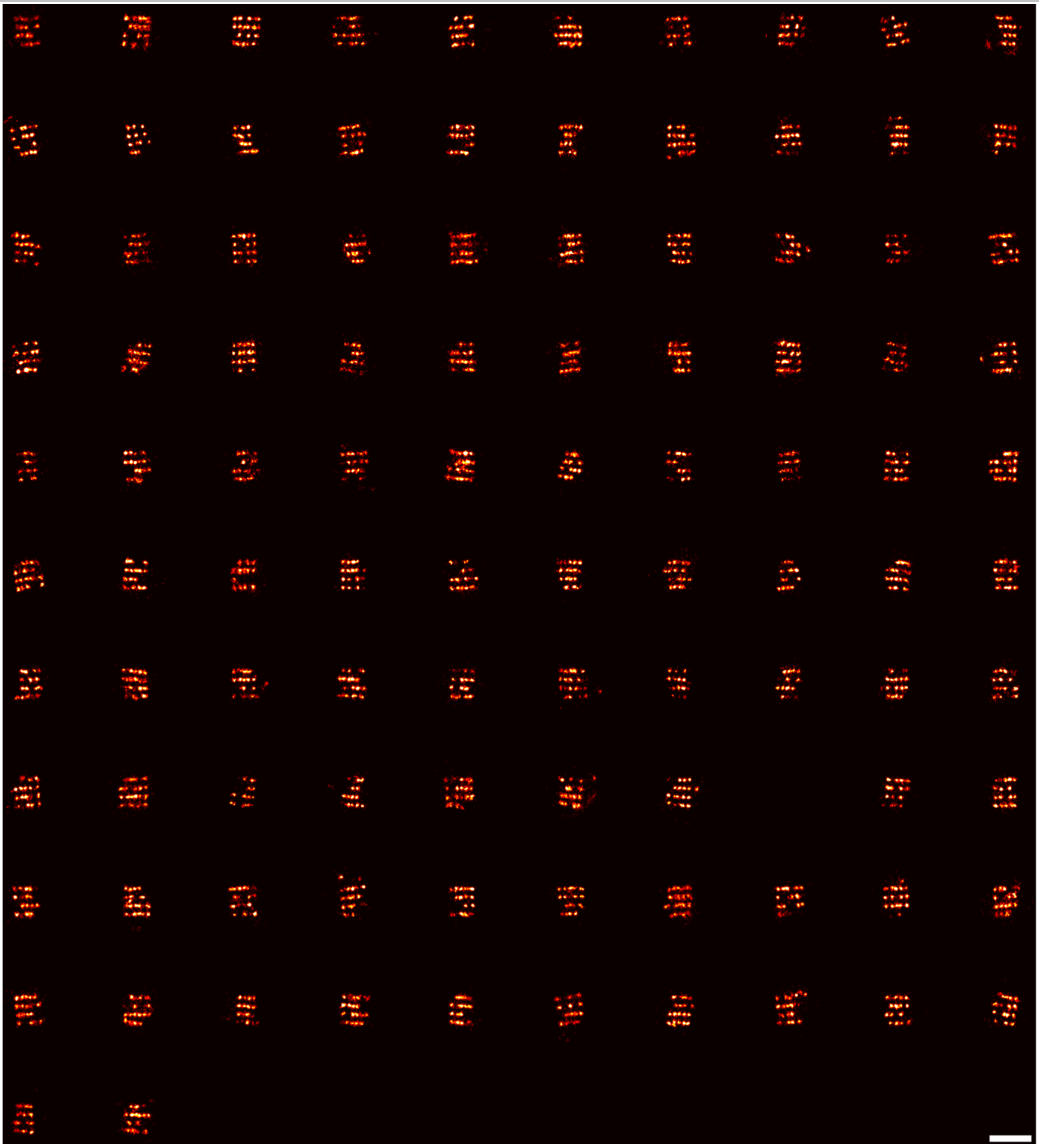

Figure S6. 20-nm-grids for pPAINT d=10nm (corresponding to **Figure 1e** (right)). Scale bar 100 nm.

Table S1. DNA origami staples extended for quantitative experiments. Incorporation values are adapted from an earlier study[2].

Staple (see Figure S1)	Sequence (See also Data S2)	Incorporation in %
Red	TTTTATTTAAGCAAATCAGATATTTTTTGT	87
Green	CATGTAATAGAATATAAAGTACCAAGCCGT	81
Blue	TATAACTAACAAAGAACGCGAGAACGCCAA	81
Yellow	CTGAGCAAAAATTAATTACATTTTGGGTTA	83

Table S2. Quantitative pPAINT measurements (corresponding to **Figure 1b**)

Distance – leash length	Exp. 1	Exp. 2	Exp. 3	Average	STD	Average scaled (with incorporation)	STD scaled (with incorporation)
Only 3'	0	1	1	1	1	**1**	**1**
Only 5'	2	1	2	2	1	**3**	**1**
3' + 5'	64	63	65	64	1	**91**	**1**

Table S3. Quantitative pPAINT measurements stem = 0 nt (corresponding **to Figure 1c**)

Distance – leash length	Exp. 1	Exp. 2	Exp. 3	Average	STD	Average scaled (with incorporation)	STD scaled (with incorporation)
0 nm – 2xT	63	63	65	64	1	**90**	**2**
5 nm – 5xT	2	1	2	2	1	**2**	**1**
10 nm – 10xT	1	1	1	1	1	**1**	**1**
0 nm – 15xT	4	1	2	2	2	**3**	**2**
5 nm – 15xT	1	3	0	1	2	**2**	**2**
10 nm – 15xT	2	1	1	1	1	**2**	**1**

Table S4. Quantitative pPAINT measurements leash length = 2xT (corresponding to **Figure 1d**)

**Distance – stem length**	**Exp. 1**	**Exp. 2**	**Exp. 3**	**Average**	**STD**	**Average scaled (with incorporation)**	**STD scaled (with incorporation)**
0 nm – 5xT	55	59	54	56	3	**79**	**4**
0 nm – 6xT	63	60	64	62	2	**88**	**3**
0 nm – 7xT	64	63	62	63	1	**89**	**1**
0 nm – 8xT	67	66	59	64	4	**91**	**6**
0 nm – 9xT	73	61	60	65	7	**92**	**10**
0 nm – 10xT	76	69	66	70	5	**100**	**7**
0 nm – 11xT	75	69	67	70	4	**100**	**6**
0 nm – 12xT	67	77	62	69	8	**97**	**11**
20 nm – 5xT	2	4	1	2	2	**3**	**2**
20 nm – 6xT	1	2	1	1	1	**2**	**1**
20 nm – 7xT	1	2	1	1	1	**2**	**1**
20 nm – 8xT	1	2	2	2	1	**2**	**1**
20 nm – 9xT	2	1	2	2	1	**2**	**1**
20 nm – 10xT	12	9	16	12	4	**18**	**5**
20 nm – 11xT	26	32	31	30	3	**42**	**5**
20 nm – 12xT	38	34	42	38	4	**54**	**6**

Table S5. Quantitative pPAINT measurements stem = 9 nt (corresponding to **Figure 1e** and **Figure S3a**)

Distance – leash length	Exp. 1	Exp. 2	Exp. 3	Average	STD	Average scaled (with incorporation)	STD scaled (with incorporation)
0 nm – 2xT	61	73	69	68	6	**96**	**9**
0 nm – 5xT	48	60	54	54	6	**77**	**9**
0 nm – 10xT	47	49	42	46	4	**65**	**5**
5 nm – 0xT	6	13	6	8	4	**12**	**6**
5 nm – 2xT	23	16	10	16	7	**23**	**9**
5 nm – 5xT	52	61	50	54	6	**77**	**8**
5 nm – 10xT	56	58	57	57	1	**81**	**1**
10 nm – 0xT	2	1	1	1	1	**2**	**1**
10 nm – 2xT	3	4	2	3	1	**4**	**1**
10 nm – 10xT	11	6	7	8	3	**11**	**4**
10 nm – 15xT	43	49	39	44	5	**61**	**7**
20 nm – 2xT	2	2	1	2	1	**2**	**1**
20 nm – 20xT	7	12	7	9	3	**12**	**4**
20 nm – 25xT	15	9	9	11	3	**15**	**5**

Table S6. Quantitative pPAINT measurements stem = 10 nt (corresponding to **Figure S3b**)

Distance – leash length	Exp. 1	Exp. 2	Exp. 3	Average	STD	Average scaled (with incorporation)	STD scaled (with incorporation)
0 nm – 2xT	76	69	66	70	5	**100**	**7**
0 nm – 5xT	59	62	66	62	4	**88**	**5**
0 nm – 10xT	55	61	61	59	3	**84**	**5**
5 nm – 0xT	41	44	47	44	3	**62**	**4**
5 nm – 2xT	65	58	65	63	4	**89**	**6**
5 nm – 5xT	59	63	67	63	4	**89**	**6**
5 nm – 10xT	55	65	62	61	5	**86**	**7**
10 nm – 0xT	2	2	4	3	1	**4**	**2**
10 nm – 2xT	12	11	7	10	3	**14**	**4**
10 nm – 10xT	54	59	53	55	3	**79**	**5**
10 nm – 15xT	68	69	60	66	5	**93**	**7**
20 nm – 2xT	12	9	16	12	4	**17**	**5**
20 nm – 20xT	23	36	26	28	7	**39**	**9**
20 nm – 25xT	36	52	36	41	9	**57**	**13**

Table S7. pPAINT 3' part

Experiment	Leash	Stem	Docking site	Distance (nm)	Staple extend (see Figure S1d)
Fig. 1b & Fig. 1c	2xT	---	GGAGAAG	0	Green – 3'
Fig. 1c	15xT	---	GGAGAAG	0	Green – 3'
Fig. 1c	2xT	---	GGAGAAG	5	Blue – 3'
Fig. 1c	15xT	---	GGAGAAG	5	Blue – 3'
Fig. 1c	2xT	---	GGAGAAG	10	Red – 3'
Fig. 1c	15xT	---	GGAGAAG	10	Red – 3'
Fig. 1d	2xT	GATAC	GGAGAAG	0	Green – 3'
Fig. 1d	2xT	CGATAC	GGAGAAG	0	Green – 3'
Fig. 1d	2xT	ACGATAC	GGAGAAG	0	Green – 3'
Fig. 1d	2xT	TACGATAC	GGAGAAG	0	Green – 3'
Fig. 1d	2xT	CTACGATAC	GGAGAAG	0	Green – 3'
Fig. 1d	2xT	GCTACGATAC	GGAGAAG	0	Green – 3'
Fig. 1d	2xT	AGCTACGATAC	GGAGAAG	0	Green – 3'
Fig. 1d	2xT	GAGCTACGATAC	GGAGAAG	0	Green – 3'
Fig. 1d	2xT	GATAC	GGAGAAG	20	Red – 3'
Fig. 1d	2xT	CGATAC	GGAGAAG	20	Red – 3'
Fig. 1d	2xT	ACGATAC	GGAGAAG	20	Red – 3'
Fig. 1d	2xT	TACGATAC	GGAGAAG	20	Red – 3'
Fig. 1d	2xT	CTACGATAC	GGAGAAG	20	Red – 3'
Fig. 1d	2xT	GCTACGATAC	GGAGAAG	20	Red – 3'
Fig. 1d	2xT	AGCTACGATAC	GGAGAAG	20	Red – 3'
Fig. 1d	2xT	GAGCTACGATAC	GGAGAAG	20	Red – 3'
Fig. 1e & (Fig. S3b) & Fig. 1f & Fig. S4-S6<br><br>() denotes 10 nt strand	2xT	(G)CTACGATAC	GGAGAAG	0	Green – 3'
Fig. 1e & (Fig. S3b)	5xT	(G)CTACGATAC	GGAGAAG	0	Green – 3'
Fig. 1e & (Fig. S3b)	10xT	(G)CTACGATAC	GGAGAAG	0	Green – 3'
Fig. 1e & (Fig. S3b)	0xT	(G)CTACGATAC	GGAGAAG	5	Blue – 3'
Fig. 1e & (Fig. S3b)	2xT	(G)CTACGATAC	GGAGAAG	5	Blue – 3'
Fig. 1e & (Fig. S3b)	5xT	(G)CTACGATAC	GGAGAAG	5	Blue – 3'
Fig. 1e & (Fig. S3b) & Fig. 1f & Fig. S4-S6	10xT	(G)CTACGATAC	GGAGAAG	5	Blue – 3'
Fig. 1e & (Fig. S3b)	0xT	(G)CTACGATAC	GGAGAAG	10	Red – 3'

Fig. 1e & (Fig. S3b)	2xT	(G)CTACGATAC	GGAGAAG	10	Red – 3’
Fig. 1e & (Fig. S3b)	10xT	(G)CTACGATAC	GGAGAAG	10	Red – 3’
Fig. 1e & (Fig. S3b) & Fig. 1f & Fig. S4-S6	15xT	(G)CTACGATAC	GGAGAAG	10	Red – 3’
Fig. 1e & (Fig. S3b)	2xT	(G)CTACGATAC	GGAGAAG	20	Red – 3’
Fig. 1e & (Fig. S3b)	20xT	(G)CTACGATAC	GGAGAAG	20	Red – 3’
Fig. 1e & (Fig. S3b)	25xT	(G)CTACGATAC	GGAGAAG	20	Red – 3’
Fig. 2	T**TTATGTTCT**T	CGAATAGTTCG	ATCTAG	---	Thiol at 5’

Table S8. pPAINT 5' part

Experiment	Docking site	Stem	Leash	Distance (nm)	Staple extend (see Figure S1d)
Fig. 1b & Fig. 1c	GAAGAGG	---	2xT	0	Red – 5'
Fig. 1c	GAAGAGG	---	15xT	0	Red – 5'
Fig. 1c	GAAGAGG	---	2xT	5	Red – 5'
Fig. 1c	GAAGAGG	---	15xT	5	Red – 5'
Fig. 1c	GAAGAGG	---	2xT	10	Green – 5'
Fig. 1c	GAAGAGG	---	15xT	10	Green – 5'
Fig. 1d	GAAGAGG	GTATC	2xT	0	Red – 5'
Fig. 1d	GAAGAGG	GTATCG	2xT	0	Red – 5'
Fig. 1d	GAAGAGG	GTATCGT	2xT	0	Red – 5'
Fig. 1d	GAAGAGG	GTATCGTA	2xT	0	Red – 5'
Fig. 1d	GAAGAGG	GTATCGTAG	2xT	0	Red – 5'
Fig. 1d	GAAGAGG	GTATCGTAGC	2xT	0	Red – 5'
Fig. 1d	GAAGAGG	GTATCGTAGCT	2xT	0	Red – 5'
Fig. 1d	GAAGAGG	GTATCGTAGCTC	2xT	0	Red – 5'
Fig. 1d	GAAGAGG	GTATC	2xT	20	Yellow – 5'
Fig. 1d	GAAGAGG	GTATCG	2xT	20	Yellow – 5'
Fig. 1d	GAAGAGG	GTATCGT	2xT	20	Yellow – 5'
Fig. 1d	GAAGAGG	GTATCGTA	2xT	20	Yellow – 5'
Fig. 1d	GAAGAGG	GTATCGTAG	2xT	20	Yellow – 5'
Fig. 1d	GAAGAGG	GTATCGTAGC	2xT	20	Yellow – 5'
Fig. 1d	GAAGAGG	GTATCGTAGCT	2xT	20	Yellow – 5'
Fig. 1d	GAAGAGG	GTATCGTAGCTC	2xT	20	Yellow – 5'
Fig. 1e & (Fig. S3b) & Fig. 1f & Fig. S4-S6	GAAGAGG	GTATCGTAG(C)	2xT	0	Red – 5'
Fig. 1e & (Fig. S3b)	GAAGAGG	GTATCGTAG(C)	5xT	0	Red – 5'
Fig. 1e & (Fig. S3b)	GAAGAGG	GTATCGTAG(C)	10xT	0	Red – 5'
Fig. 1e & (Fig. S3b)	GAAGAGG	GTATCGTAG(C)	0xT	5	Red – 5'
Fig. 1e & (Fig. S3b)	GAAGAGG	GTATCGTAG(C)	2xT	5	Red – 5'
Fig. 1e & (Fig. S3b)	GAAGAGG	GTATCGTAG(C)	5xT	5	Red – 5'
Fig. 1e & (Fig. S3b) & Fig. 1f & Fig. S4-S6	GAAGAGG	GTATCGTAG(C)	10xT	5	Red – 5'
Fig. 1e & (Fig. S3b)	GAAGAGG	GTATCGTAG(C)	0xT	10	Green – 5'
Fig. 1e & (Fig. S3b)	GAAGAGG	GTATCGTAG(C)	2xT	10	Green – 5'
Fig. 1e & (Fig. S3b)	GAAGAGG	GTATCGTAG(C)	10xT	10	Green – 5'
Fig. 1e & (Fig. S3b) & Fig. 1f & Fig. S4-S6	GAAGAGG	GTATCGTAG(C)	15xT	10	Green – 5'
Fig. 1e & (Fig. S3b)	GAAGAGG	GTATCGTAG(C)	2xT	20	Yellow – 5'
Fig. 1e & (Fig. S3b)	GAAGAGG	GTATCGTAG(C)	20xT	20	Yellow – 5'
Fig. 1e & (Fig. S3b)	GAAGAGG	GTATCGTAG(C)	25xT	20	Yellow – 5'
Fig. 2	ATACAA	CGAACTATTCG	T**TCAATGTAT**T	---	Thiol at 3'

Table S9. Imager sequences

Imager name	Sequence (5' to 3')	5'-mod	3'-mod	Vendor
pPS*	TCTCCTTCCTCT	None	Cy3B	MetaBion
pPJL*	CTAGATTTTTGTAT	None	Cy3B	MetaBion
P3*	TAATGAAGA	None	Cy3B	MetaBion
P5*	ATACATTGA	None	Cy3B	MetaBion
P6*	TTTACCTAA	None	Cy3B	MetaBion
P39*	AGAACATAA	None	Cy3B	MetaBion

Table S10. List of biotinylated DNA staple strands

No	Position	Name	Sequence	Mod
1	C02	18[63]20[56]BIOTIN	ATTAAGTTTACCGAGCTCGAATTCGGGAAACCTGTCGTGC	5'-BT
2	C09	4[63]6[56]BIOTIN	ATAAGGGAACCGGATATTCATTACGTCAGGACGTTGGGAA	5'-BT
3	G02	18[127]20[120]BIOTIN	GCGATCGGCAATTCCACACAACAGGTGCCTAATGAGTG	5'-BT
4	G09	4[127]6[120]BIOTIN	TTGTGTCGTGACGAGAAACACCAAATTTCAACTTTAAT	5'-BT
5	K02	18[191]20[184]BIOTIN	ATTCATTTTTGTTTGGATTATACTAAGAAACCACCAGAAG	5'-BT
6	K09	4[191]6[184]BIOTIN	CACCCTCAGAAACCATCGATAGCATTGAGCCATTTGGGAA	5'-BT
7	O02	18[255]20[248]BIOTIN	AACAATAACGTAAAACAGAAATAAAAATCCTTTGCCCGAA	5'-BT
8	O09	4[255]6[248]BIOTIN	AGCCACCACTGTAGCGCGTTTTCAAGGGAGGGAAGGTAAA	5'-BT

Table S11. Handle sequences

Handle Name	Sequence (5' to 3')	5'-mod	3'-mod	Vendor
pPS-3'	GGAGAAG	Stem	None	IDT
pPS-5'	GAAGAGG	None	Stem	IDT
pPJL-3'	ATCTAG	Stem	None	IDT
pPJL-5'	ATACAA	None	Stem	IDT
P3	TCTTCATTA	Staple DNA origami	None	IDT
P5	TCAATGTAT	Leash	Leash	IDT
P6	TTAGGTAAA	Staple DNA origami	None	IDT
P39	TTATGTTCT	Leash	Leash	IDT

Table S12. Imaging parameters

Dataset	Parameters	Buffer	Intensity
**Figure 1b – e & Figure S3**	Round 1: 100 ms, 20k Frames, 7.5 nM (pPS), 2.5 nM (P3)<br>Round2 : 100 ms, 5k Frames, 2.5 nM (P3), 2.5 nM (P6)	B + Trolox, POC<br>B + Trolox, PCA, PCD	11 W/cm^2
**Figure 1f & Figure S4-S6**	200 ms, 20k Frames, 5 nM (pPS)	B + Trolox, PCA, PCD	564 W/cm^2
**Figure 2**	50 ms, 20k Frames, 0.3 nM (P5), 0.3 nM (P39) and 2 nM (pPJL)	C + Trolox, PCA, PCD	508 W/cm^2

References

[1] J. Schnitzbauer, M. T. Strauss, T. Schlichthaerle, F. Schueder, R. Jungmann, Nat Protoc 2017, 12, 1198-1228.
[2] M. T. Strauss, F. Schueder, D. Haas, P. C. Nickels, R. Jungmann, Nat Commun 2018, 9, 1600.

Author Contributions

F.S. and J.L. contributed equally. F.S. conceived and performed experiments, analyzed data and contributed to the writing of the manuscript. J.L. conceived and performed experiments, analyzed data, wrote the manuscript. D.H. performed experiments and developed data analysis. K.S.B. designed experiments. P.Y. supervised initial experiments. J.E. developed cell lines and supervised part of the study. R.J. conceived and supervised the study, analyzed and interpreted data, and wrote the manuscript. All authors reviewed and approved the manuscript.
